# Supplementary material for: Spatial Variation in Excess Mortality Across Europe: A Cross-Sectional Study of 561 Regions in 21 Countries
Source: J Epidemiol Glob Health. 2024 Feb 20;14(2):470–9. doi: 10.1007/s44197-024-00200-0 (PMC11176282; doi:10.1007/s44197-024-00200-0)
Supplement: Supplementary file 1 — Supplementary file1 (DOCX 1857 KB) [file 44197_2024_200_MOESM1_ESM.docx]

**Spatial variation in excess mortality across Europe: a cross-sectional study of 561 regions in 21 countries**

**Supplementary Information**

Florian Bonnet^[[1]](#footnote-1)^, Pavel Grigoriev, Markus Sauerberg, Ina Alliger, Michael Mühlichen, Carlo-Giovanni Camarda

# Online Supplementary Information A

**Table A1: Regional division, sources, data information and adjustments by country**

| **Country** | **Spatial units** | **Source** | **Period** | **Upper age limit** | **Adjustments/comments** |
| --- | --- | --- | --- | --- | --- |
| Austria | 9 NUTS 2 units (‘Länder’) | Statistics Austria | 1990–2020 | Deaths: 90  Pop.: 100 | – |
| Belgium | 11 NUTS 2 units (‘provincies’) | Belgian Statistical Office | 1992–2020 | Deaths: 100  Pop.: 100 | – |
| Czechia | 14 NUTS 3 units (‘kraje’) | Czech Statistical Office | 1996–2020 | Deaths: 95  Pop.: 95 | – |
| Denmark | 11 NUTS 3 units (‘landsdele’) | Statistics Denmark | 2008–2020 | Deaths: 100  Pop.: 100 | – |
| France | 95 NUTS 3 units (‘départements’) | INSEE | 1970–2020 | Deaths: 105  Pop.: 105 (after 2016: 95) | Non-European areas excluded; north and south of Corse merged to maintain consistent time series. |
| Germany | 96 ROR (‘Raumordnungs-regionen’) | Statistical Offices of the German Länder | 1992–2020 | Deaths: 90  Pop.: 90 | Harmonised to apply current territorial administrative division (as of Dec 2022) to the whole study period and to eliminate the Census 2011 break; 400 NUTS 3 units (‘Kreise’) aggregated to 96 ROR units according to classification of BBSR (2017). See (38) for more details. |
| Hungary | 8 NUTS 2 units (‘tervezési-statisztikai régiók’) | Hungarian Central Statistical Office, Eurostat | 2001–2020 | Deaths: 90  Pop.: 90 | – |
| Iceland | 1 NUTS 2 unit | Human Mortality Database | 1970–2020 | Deaths: 110  Pop.: 110 | – |
| Ireland | 1 NUTS 1 unit | Human Mortality Database | 1990–2020 | Deaths: 110  Pop.: 110 | No subnational division due to data availability issues |
| Italy | 92 NUTS 3 units (‘province’) | ISTAT | 1995–2020 | Deaths: 100  Pop.: 100 | We merged the following regions to maintain a consistent time series: 1) Biella + Vercelli, 2) Novara + Verbano, 3) Como + Lecco, 4) Milano + Lodi + Monza + Brianza, 5) Rimini + Forli-Cesena, 5) Firenze + Prato, 6) Cagliari + Medio Campidano + Carbonia-Iglesias + Ogliastra + Oristano + Nuoro, 7) Sassari + Olbia-Tempio, 8) Foggia + Bari + Barletta, 9) Fermo + Ascoli-Piceno, 10) Crotone + Vibo Valentia + Cantanzaro. |
| Luxembourg | 1 NUTS 3 unit | Human Mortality Database | 1996–2020 | Deaths: 110  Pop.: 110 | – |
| Netherlands | 11 NUTS 2 units (‘provincies’) | Statistics Netherlands | 2001–2020 | Deaths: 100  Pop.: 100 | – |
| Norway | 7 NUTS 2 units (‘landsdeler’) | Statistics Norway | 2000–2020 | Deaths: 100  Pop.: 105 | Harmonised to apply current territorial administrative divisions; we excluded remote islands Svalbard and Jan Mayen |
| Poland | 73 NUTS 3 units (‘podregiony’) | Statistics Poland | 2006–2020 | Deaths: 85  Pop.: 85 (until 2005: 65) | – |
| Portugal | 5 NUTS 2 units (‘regiões’) | National Institute of Statistics, Eurostat | 1992–2020 | Deaths: 85  Pop.: 95 | For visibility reasons, we excluded the remote islands Azores and Madeira. |
| Slovakia | 8 NUTS 3 units (‘kraje’) | Slovakian Statistical Office | 1996–2020 | Deaths: 85  Pop.: 110 | – |
| Slovenia | 2 NUTS 2 units (‘kohezijske regije’) | Slovenian Statistical Office | 2002–2020 | Deaths: 100  Pop.: 100 | – |
| Spain | 50 NUTS 3 units (‘provincias’) | National Statistics Institute | 1990–2020 | Deaths: 100  Pop.: 100 (until 2001:85) | For visibility reasons, we excluded the Canary Islands. |
| Sweden | 21 NUTS 3 units (‘län’) | Statistics Sweden | 1969–2020 | Deaths: 100  Pop.: 100 | – |
| Switzerland | 7 NUTS 2 units (‘Grossregionen’) | Federal Statistical Office | 1991–2020 | Deaths: 95  Pop.: 100 | – |
| United Kingdom | 37 NUTS 2 regions | Office for National Statistics (for England & Wales); Human Mortality Database (for Northern Ireland & Scotland) | 2002–2020 | England & Wales  Deaths: 90  Pop.: 90  Northern Ireland & Scotland  Deaths: 110  Pop.: 110 | NUTS 1 level data for Northern Ireland and Scotland due to data quality issues |

**Table A2: Comparison of estimated life expectancy changes with previous studies**

| **Country** | **Difference in e0, females** | | |  | **Difference in e0, males** | | |
| --- | --- | --- | --- | --- | --- | --- | --- |
|  | **Aburto et al. 2022** | **Islam et al. 2021** | **This paper** |  | **Aburto et al. 2022** | **Islam et al. 2021** | **This paper** |
| **Austria** | -0.67 (-0.83,-0.53) | -0.62 (-0.75,-0.51) | -0.55 (-0.71,-0.39) |  | -0.81 (-0.98,-0.65) | -0.84 (-0.96,-0.72) | -0.80 (-0.98,-0.63) |
| **Belgium** | -1.14 (-1.29,-1.01) | -1.11 (-1.26,-0.96) | -0.92 (-1.03,-0.80) |  | -1.19 (-1.33,-1.05) | -1.31 (-1.42,-1.20) | -1.14 (-1.27,-1.02) |
| **Bulgaria** | -1.29 (-1.45,-1.10) | -1.37 (-1.74,-1.01) | NA |  | -1.60 (-1.81,-1.41) | -1.96 (-2.11,-1.81) | NA |
| **Switzerland** | -0.65 (-0.80,-0.47) | -0.57 (-0.71,-0.43) | -0.53 (-0.69,-0.38) |  | -0.97 (-1.14,-0.81) | -1.05 (-1.28,-0.83) | -1.03 (-1.20,-0.86) |
| **Chile** | -0.85 (-1.01,-0.68) | -0.88 (-1.28,-0.50) | NA |  | -1.27 (-1.42,-1.14) | -1.64 (-1.97,-1.32) | NA |
| **Czech Rep.** | -0.90 (-1.05,-0.75) | -0.94 (-1.09,-0.79) | -0.94 (-1.06,-0.82) |  | -1.10 (-1.24,-0.95) | -1.26 (-1.36,-1.15) | -1.20 (-1.36,-1.03) |
| **Germany** | -0.23 (-0.28,-0.18) | -0.17 (-0.46,+0.12) | -0.16 (-0.23,-0.09) |  | -0.38 (-0.44,-0.32) | -0.46 (-0.67,-0.25) | -0.31 (-0.39,-0.24) |
| **Denmark** | +0.07 (-0.14,+0.28) | -0.09 (-0.2,+0.03) | +0.02 (-0.15,+0.18) |  | +0.05 (-0.14,+0.27) | -0.09 (-0.19,+0.00) | -0.01 (-0.18,+0.17) |
| **Estonia** | -0.43 (-0.87,-0.02) | -0.52 (-0.61,-0.43) | NA |  | -0.41 (-0.90,+0.05) | -0.75 (-1.00,-0.50) | NA |
| **Spain** | -1.50 (-1.58,-1.43) | -1.13 (-1.37,-0.90) | -1.13 (-1.21,-1.05) |  | -1.44 (-1.51,-1.36) | -1.35 (-1.53,-1.18) | -1.36 (-1.45,-1.26) |
| **Finland** | +0.01 (-0.19,+0.21) | -0.03 (-0.13,+0.06) | NA |  | -0.14 (-0.39,+0.07) | -0.34 (-0.42,-0.25) | NA |
| **France** | -0.60 (-0.66,-0.54) | -0.67 (-0.84,-0.51) | -0.49 (-0.57,-0.41) |  | -0.67 (-0.74,-0.61) | -0.53 (-0.66,-0.40) | -0.76 (-0.86,-0.68) |
| **Eng. and W.** | -0.91 (-0.98,-0.85) | -0.80 (-0.99,-0.62) | NA |  | -1.14 (-1.21,-1.08) | -1.20 (-1.35,-1.04) | NA |
| **N. Ireland** | -0.81 (-1.14,-0.43) | -0.62 (-0.85,-0.40) | -0.80 (-1.09,-0.52) |  | -0.76 (-1.14,-0.36) | -1.07 (-1.39,-0.76) | -0.92 (-1.23,-0.61) |
| **Scotland** | -0.51 (-0.73,-0.28) | -0.54 (-0.71,-0.36) | -0.45 (-0.63,-0.27.) |  | -1.06 (-1.28,-0.85) | -1.24 (-1.45,-1.03) | -0.98 (-1.19,-0.77) |
| **Greece** | -0.23 (-0.37,-0.08) | -0.34 (-0.55,-0.15) | NA |  | -0.36 (-0.52,-0.19) | -0.46 (-0.61,-0.30) | NA |
| **Croatia** | -0.83 (-1.10,-0.61) | -0.72 (-0.92,-0.51) | NA |  | -0.83 (-1.07,-0.58) | -0.92 (-1.09,-0.75) | NA |
| **Hungary** | -0.65 (-0.78,-0.51) | -0.79 (-0.94,-0.64) | -0.73 (-0.88,-0.58) |  | -0.71 (-0.87,-0.55) | -0.97 (-1.07,-0.87) | -0.88 (-1.05,-0.70) |
| **Iceland** | -0.07 (-0.95,+0.90) | +0.05 (-0.34,+0.44) | +0.00 (-0.59,+0.58) |  | -0.20 (-1.18,+0.84) | -0.26 (-0.74,+0.19) | -0.22 (-0.90,+0.46) |
| **Italy** | -1.01 (-1.07,-0.95) | -1.05 (-1.33,-0.77) | -0.85 (-0.93,-0.78) |  | -1.25 (-1.31,-1.18) | -1.56 (-1.80,-1.34) | -1.36 (-1.45,-1.28) |
| **Lithuania** | -1.26 (-1.54,-0.97) | -1.21 (-1.36,-1.05) | NA |  | -1.69 (-2.00,-1.36) | -1.83 (-2.07,-1.59) | NA |
| **Netherlands** | -0.57 (-0.68,-0.46) | -0.66 (-0.76,-0.56) | -0.46 (-0.56,-0.36) |  | -0.80 (-0.92,-0.68) | -1.03 (-1.14,-0.92) | -0.89 (-1.00,-0.78) |
| **Norway** | +0.18 (-0.04,+0.40) | +0.07 (-0.01,+0.14) | -0.05 (-0.20,+0.10) |  | +0.18 (-0.04,+0.42) | +0.06 (+0.00,+0.13) | -0.26 (-0.44,-0.09) |
| **Poland** | -1.07 (-1.15,-1.00) | -1.02 (-1.16,-0.89) | -1.04 (-1.13,-0.95) |  | -1.50 (-1.58,-1.41) | -1.54 (-1.67,-1.41) | -1.57 (-1.68,-1.46) |
| **Portugal** | -0.69 (-0.84,-0.54) | -0.78 (-0.94,-0.61) | -0.60 (-0.72,-0.48) |  | -0.83 (-1.02,-0.67) | -0.93 (-1.03,-0.84) | -0.76 (-0.92,-0.60) |
| **Sweden** | -0.59 (-0.76,-0.46) | -0.54 (-0.63,-0.45) | -0.30 (-0.41,-0.20) |  | -0.87 (-1.02,-0.72) | -0.90 (-0.99,-0.81) | -0.72 (-0.83,-0.60) |
| **Slovenia** | -1.06 (-1.35,-0.71) | -1.02 (-1.18,-0.86) | -0.91 (-1.15,-0.68) |  | -0.94 (-1.29,-0.57) | -1.04 (-1.21,-0.88) | -1.08 (-1.35,-0.82) |
| **Slovakia** | -0.79 (-0.99,-0.58) | -0.85 (-1.00,-0.70) | -0.75 (-0.95,-0.54) |  | -0.83 (-1.07,-0.59) | -1.00 (-1.12,-0.88) | -0.91 (-1.14,-0.68) |
| **USA** | -1.65 (-1.68,-1.62) | -1.61 (-1.70,-1.51) | NA |  | -2.23 (-2.26,-2.20) | -2.27 (-2.39,-2.15) | NA |
| **Luxembourg** | NA | -0.55 (-0.84,-0.28) | -0.55 (-1.01,-0.08) |  | NA | -0.35 (-0.64,-0.07) | -0.69 (-1.21,-0.17) |
| **Ireland** | NA | NA | -0.36 (-0.54,-0.19) |  | NA | NA | -0.27 (-0.46,-0.08) |


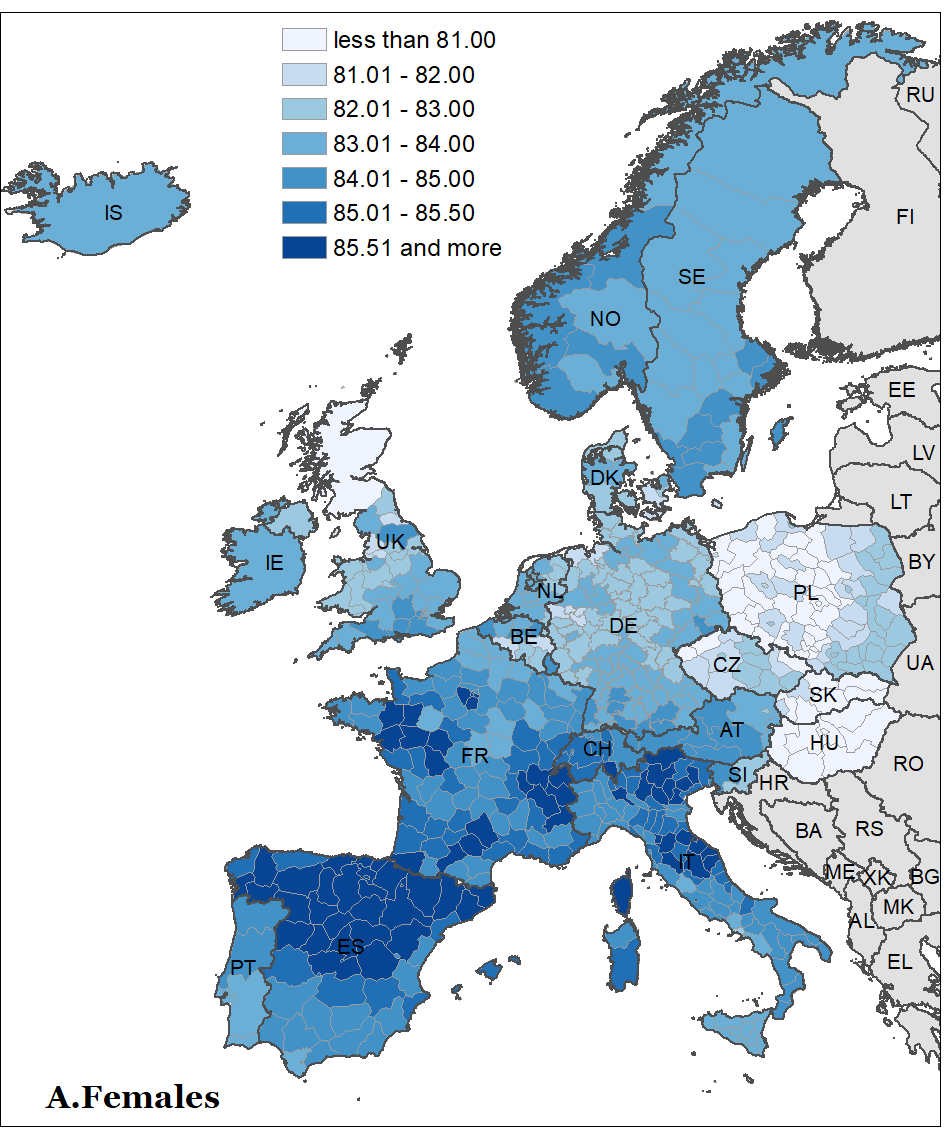

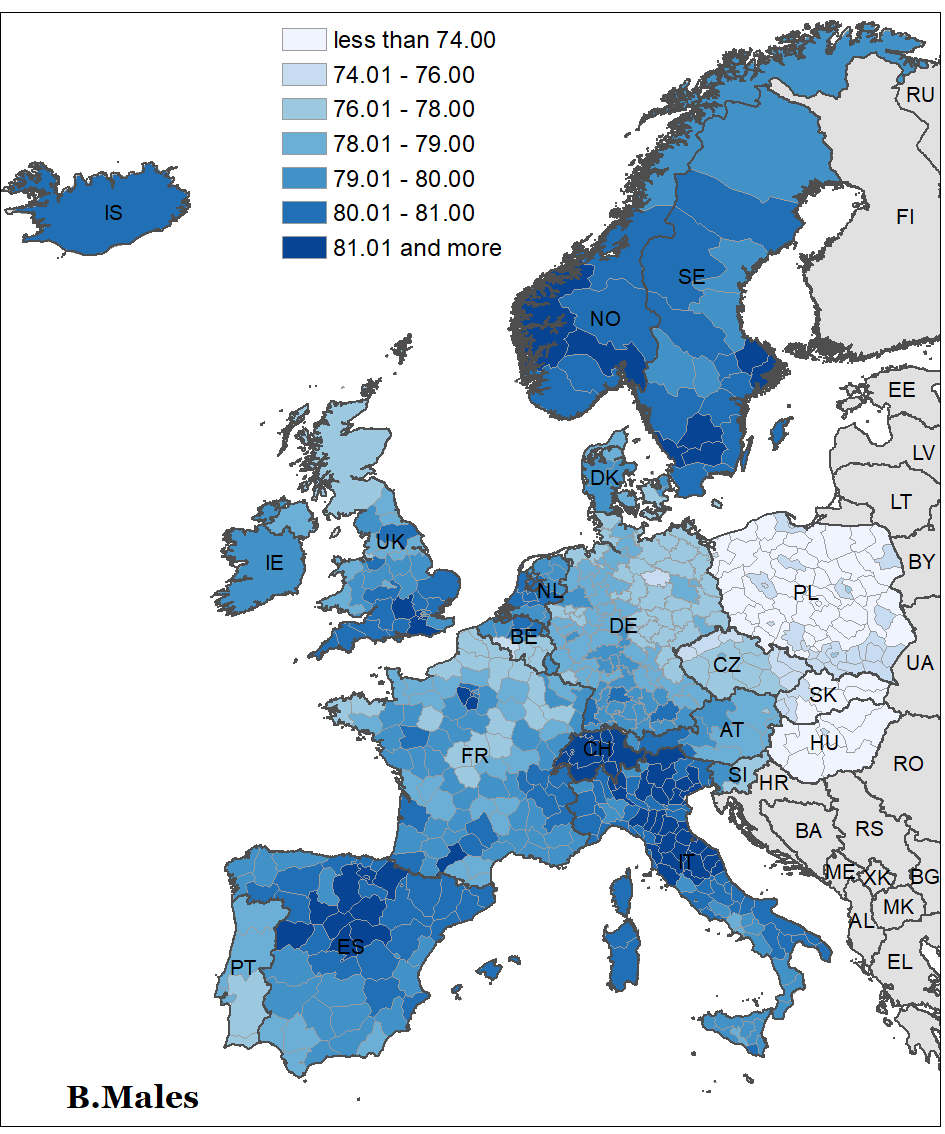


**Figure A1. Life expectancy at birth across 561 spatial units in 21 European countries, 2015–2019**


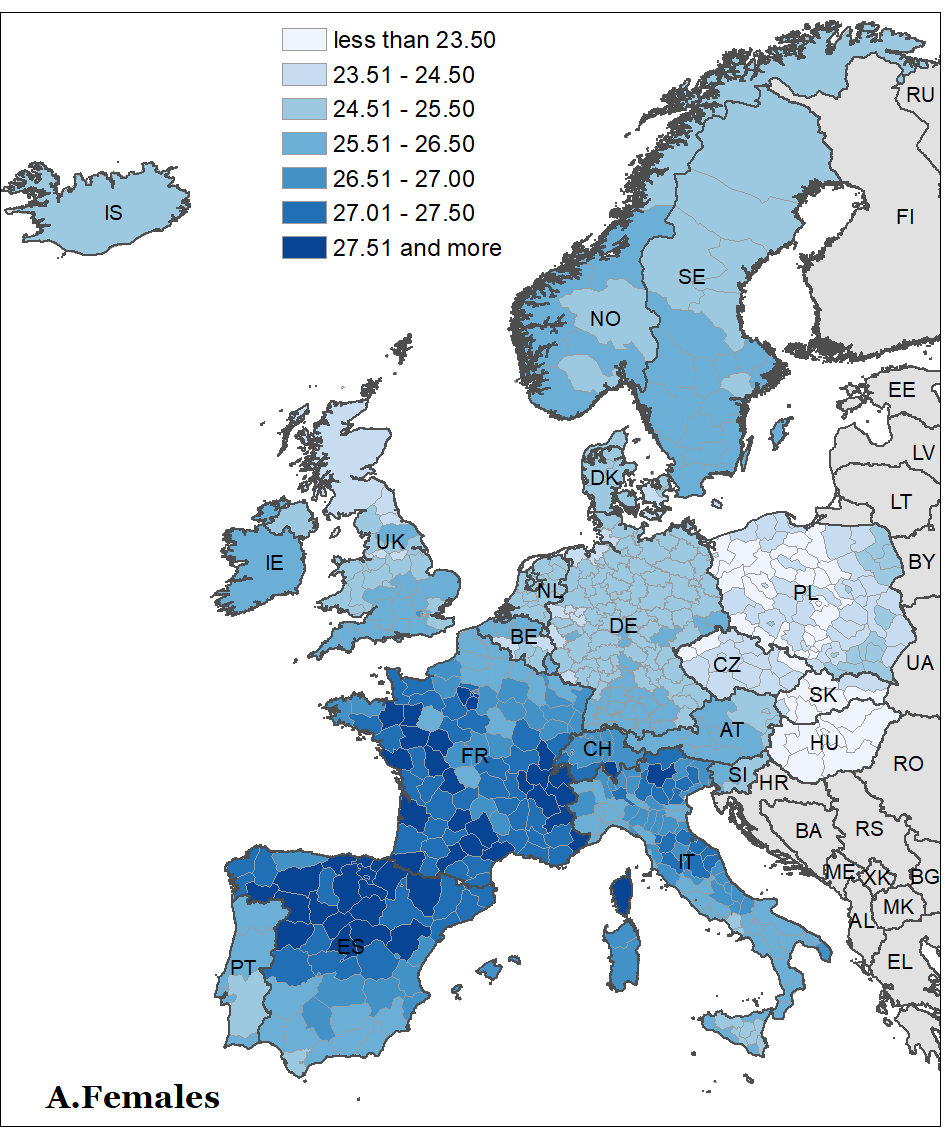

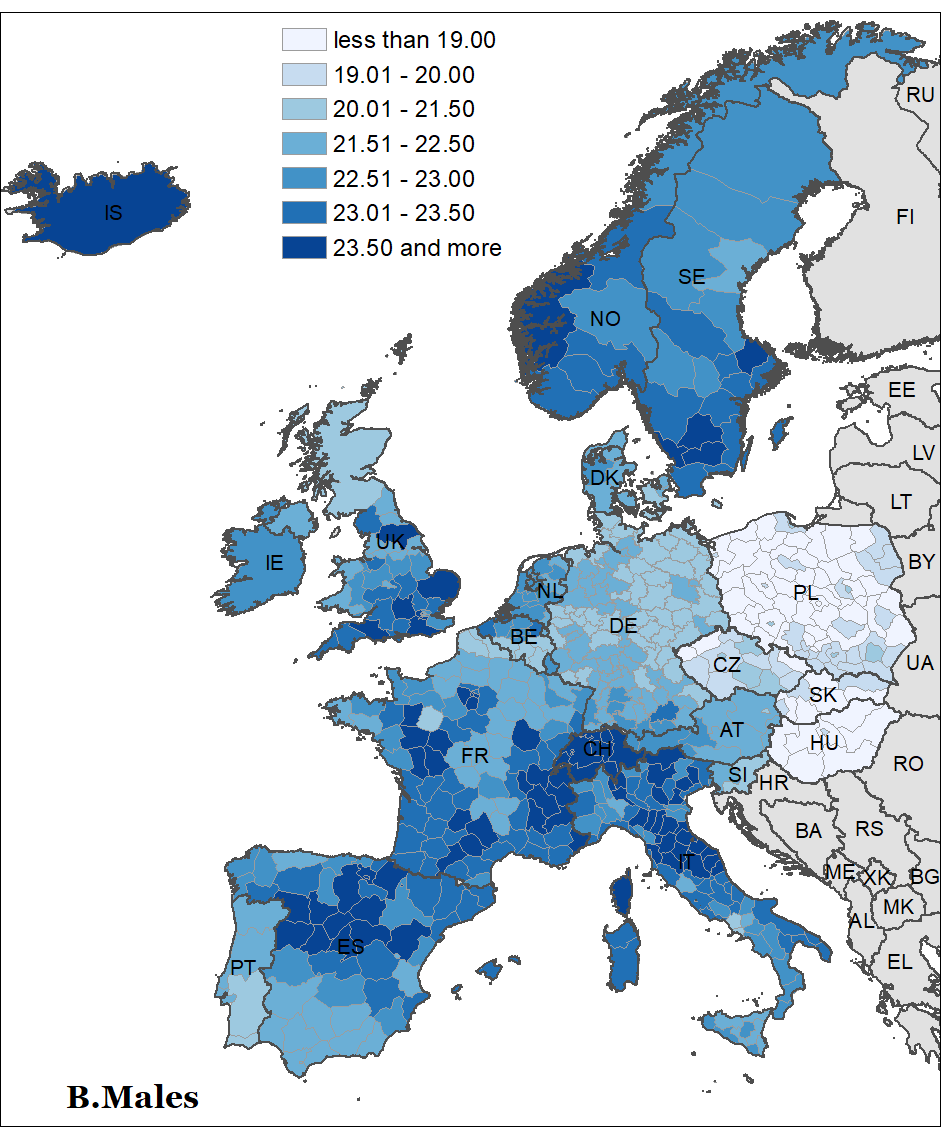


**Figure A2. Life expectancy at age 60 across 561 spatial units in 21 European countries, 2015–2019**


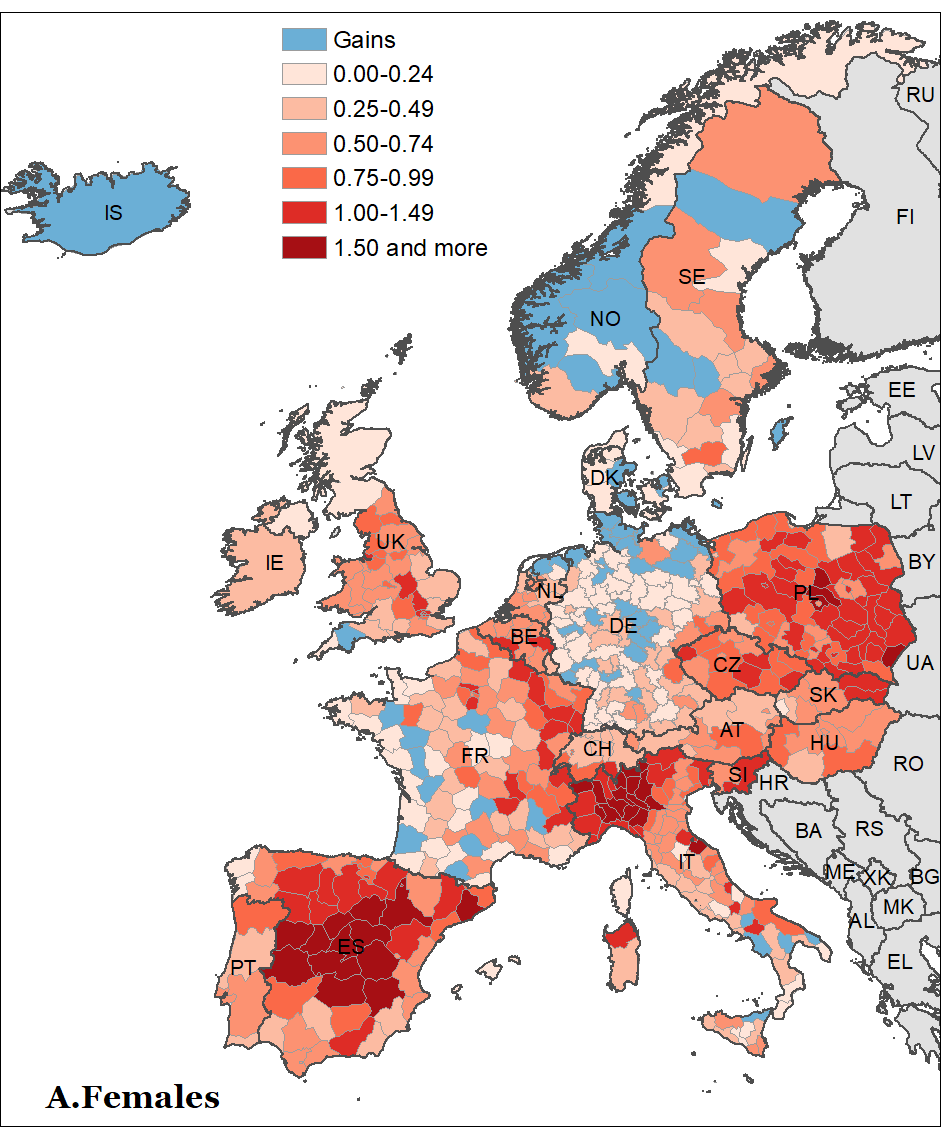

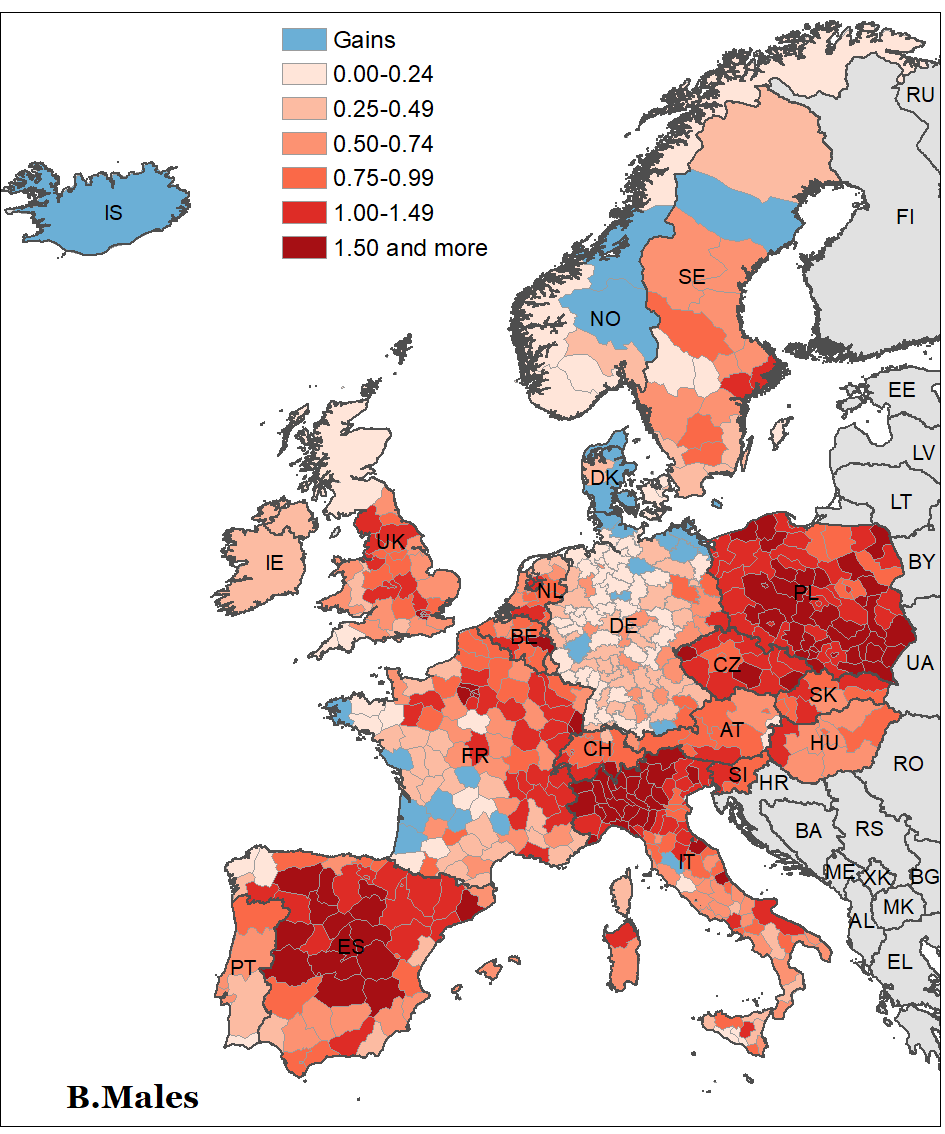


**Figure A3. Spatial distribution of losses of life expectancy at age 60 across 21 European countries in 2020**


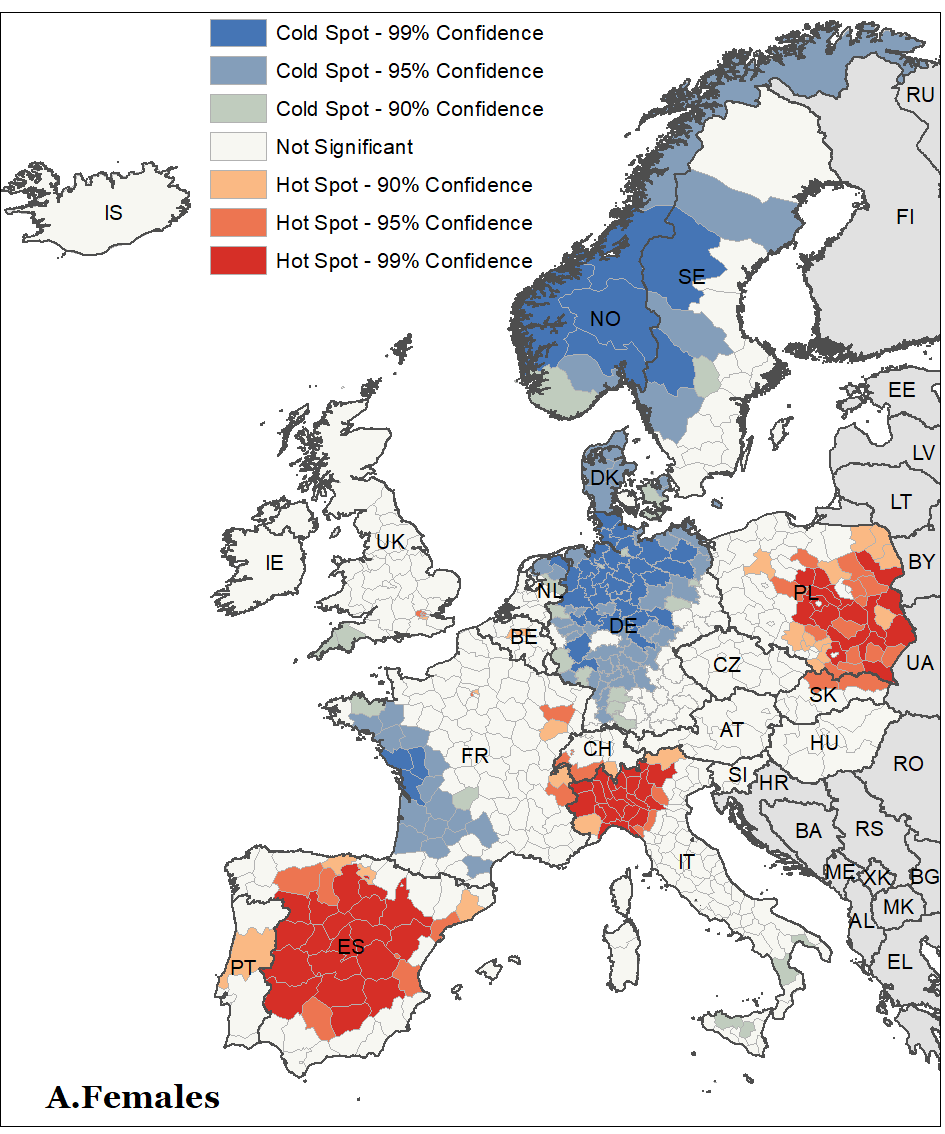

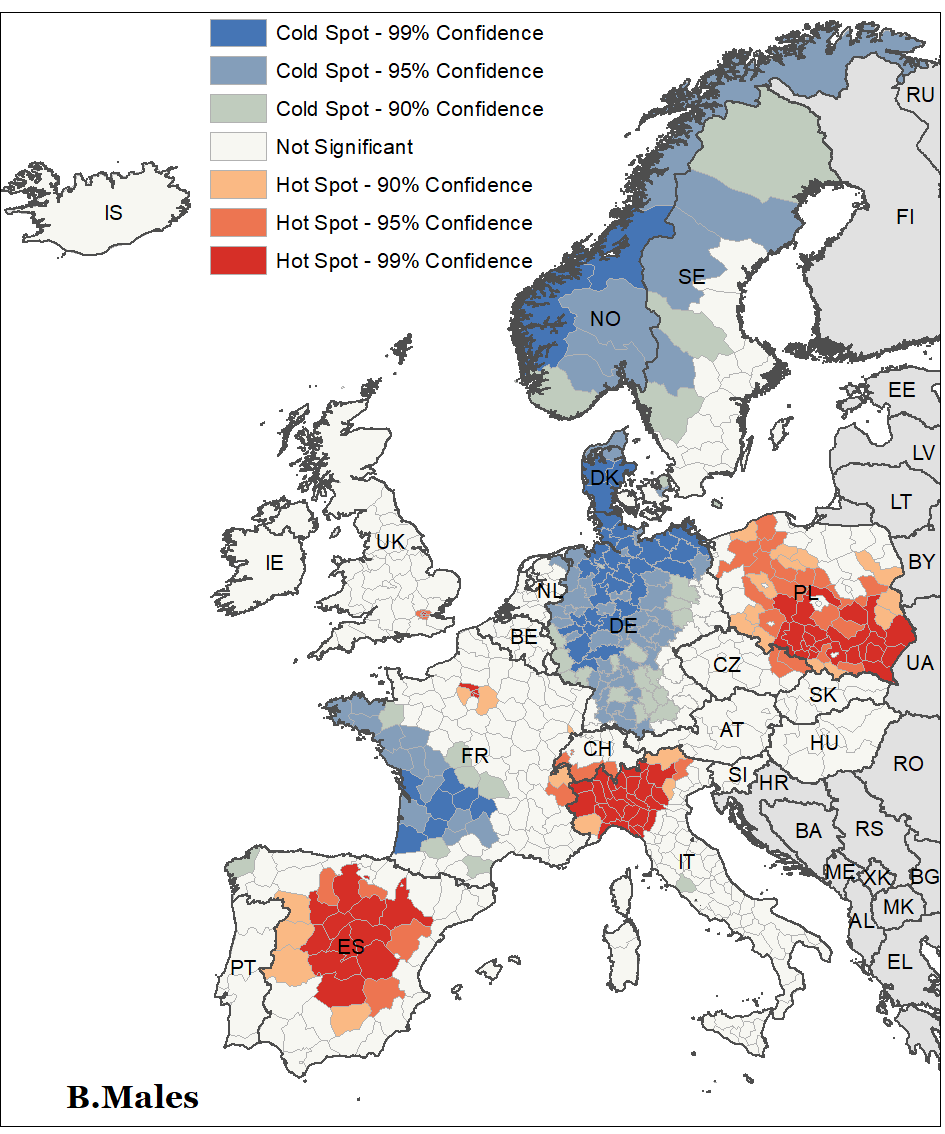


**Figure A4. Hot and cold spots of losses of life expectancy at age 60 across 21 European countries in 2020**

**
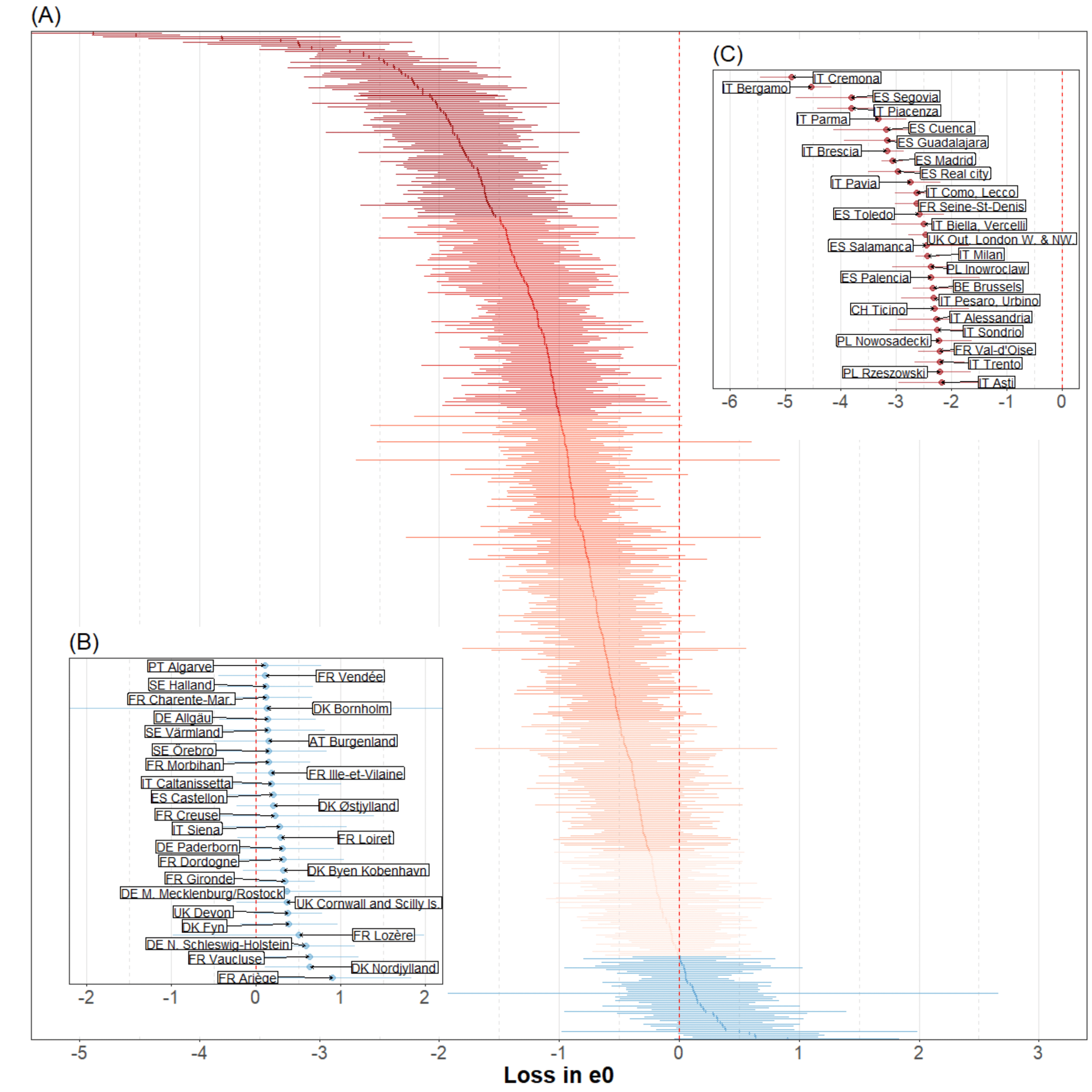
Figure A5. Losses or gains of life expectancy at birth (e0) associated with COVID-19 pandemic across 561 spatial units in 21 European countries, 2020, males.**

**
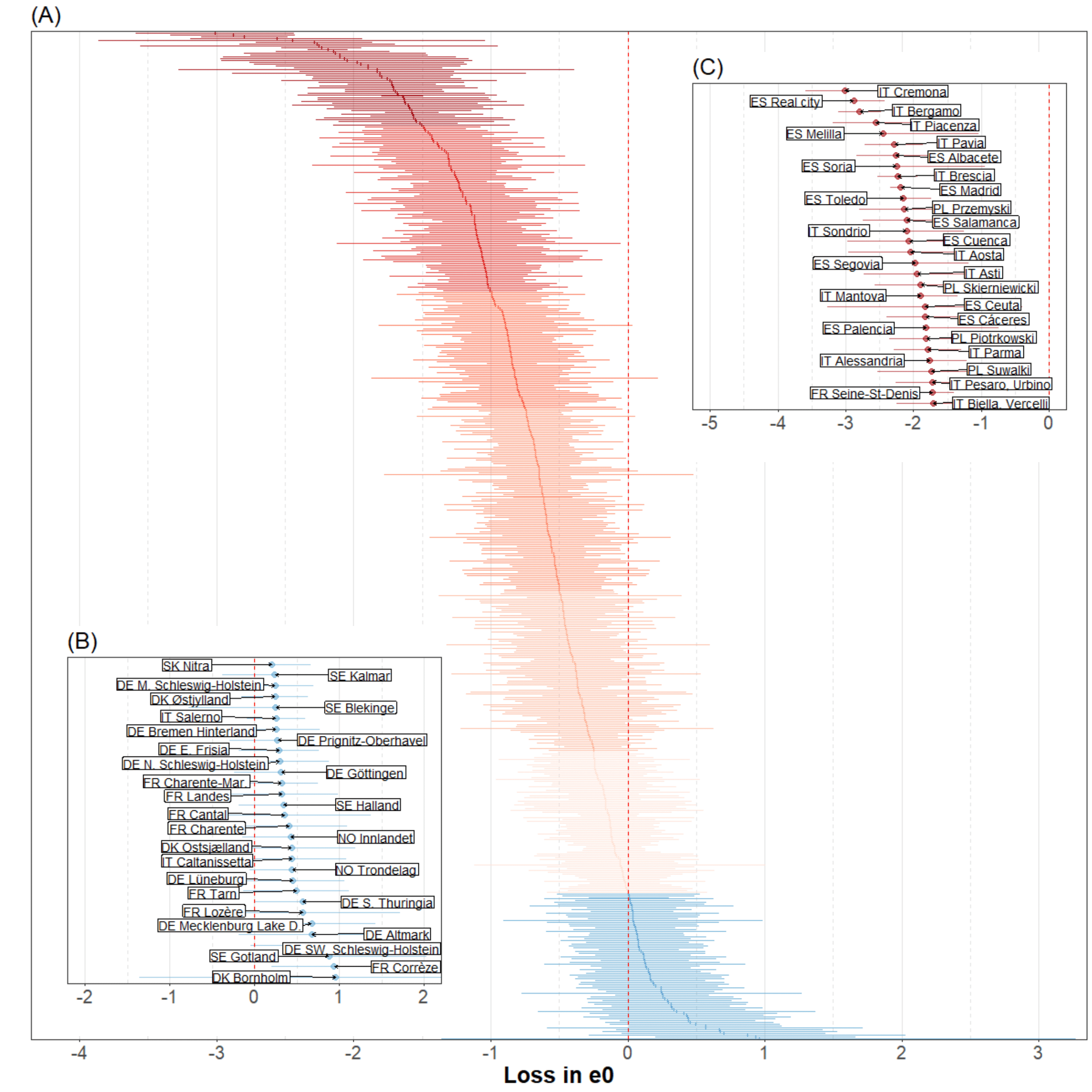
**

**Figure A6. Losses or gains of life expectancy at birth (e0) associated with COVID-19 pandemic across 561 spatial units in 21 European countries, 2020, females.**

# Online Supplementary Information B

Detailed values of our estimates and data visualisation tool are available at:

<https://osf.io/h68wz/?view_only=47353f6f4b2e41cab3c761246e59d615>

Please read first “Online Appendix B.pdf”.

# Online Supplementary Information C

A detailed description of the analytic procedure to compute excess mortality is available at:

<https://osf.io/h68wz/?view_only=47353f6f4b2e41cab3c761246e59d615>

1. Corresponding author: florian.bonnet@ined.fr, 9 cours des Humanités, 93300 Aubervilliers, France. [↑](#footnote-ref-1)
